# Visit‐to‐visit systolic blood pressure variability, blood pressure treatment intensity, and cognitive decline

**DOI:** 10.1002/alz.71726

**Published:** 2026-07-31

**Authors:** Yue Qiao, Zihan Sun, Xunming Ji, Hugh S Markus, Wenbo Zhao

**Affiliations:** ^1^ Department of Neurology Xuanwu Hospital Capital Medical University, National Centre for Neurological Disorders Beijing China; ^2^ Stroke Research Group, Department of Clinical Neurosciences University of Cambridge Cambridge UK; ^3^ Beijing Institute for Brain Disorders Capital Medical University Beijing China

**Keywords:** blood pressure control, blood pressure variability, cognitive decline, diabetes mellitus, hypertension

## Abstract

**INTRODUCTION:**

Visit‐to‐visit blood pressure variability (BPV) may be associated with cognitive decline beyond mean blood pressure (BP), but its relevance across treatment contexts remains uncertain.

**METHODS:**

We pooled individual‐participant data from Action to Control Cardiovascular Risk in Diabetes Memory in Diabetes (ACCORD‐MIND) and Systolic Blood Pressure Intervention Trial Memory and Cognition in Decreased Hypertension (SPRINT‐MIND) (*n* = 11,104). Participants had baseline and follow‐up cognitive testing and ≥3 BP measurements from 3 months onwards. The primary exposure was systolic BP variation independent of the mean (SBP‐VIM). The primary outcome was annualized change in standardized Digit Symbol Substitution or Coding Test Z‐scores.

**RESULTS:**

Each 10% increment in SBP‐VIM was independently associated with faster annual cognitive decline (*β* = −0.008 per year; 95% confidence interval [CI]: −0.014 to −0.003) after adjustment including mean SBP. Associations were observed in the intensive but not standard BP treatment arms of both trials, although the pooled interaction was not statistically significant (*p* = 0.054).

**DISCUSSION:**

Higher systolic BPV was associated with accelerated cognitive decline independently of mean BP. The exploratory treatment‐context pattern warrants prospective confirmation.

## Highlights

- Higher systolic blood pressure (SBP) variability is linked to faster cognitive decline.
- Association persists after adjusting for mean SBP and cardiometabolic factors.
- Effect appeared stronger under intensive BP lowering, an exploratory pattern.
- Targeting BP variability may complement mean BP control to preserve cognitive function.

## 1 BACKGROUND

Hypertension is one of the most well‐established, modifiable risk factors for cognitive decline and dementia, and antihypertensive therapy has been investigated as a potential strategy to mitigate this risk.^1^ Although large randomized trials have shown that blood pressure (BP) lowering reduces cardiovascular events,^2^, ^3^ its cognitive effects appear to differ across cardiometabolic risk profiles. For example, the SPRINT‐MIND (Systolic Blood Pressure Intervention Trial Memory and Cognition in Decreased Hypertension) trial, which enrolled hypertensive patients without diabetes, demonstrated that intensive systolic BP (SBP) lowering to <120 mmHg reduced the risk of cognitive impairment compared with a standard target of <140 mmHg.^4^ By contrast, the ACCORD‐MIND (Action to Control Cardiovascular Risk in Diabetes Memory in Diabetes) trial, which included patients with type 2 diabetes, showed no significant cognitive benefit from intensive SBP lowering despite a similar antihypertensive strategy.^5^ The mechanisms underlying this disparity remain uncertain, but these findings underscore the need to explore factors beyond mean SBP that may influence cognitive outcomes, especially in patients with diabetes.

Beyond mean BP levels, visit‐to‐visit blood pressure variability (BPV) reflects hemodynamic instability and vascular stress. Higher BPV has been associated with cardiovascular events, stroke, and all‐cause mortality.^6^ Emerging evidence from observational studies and meta‐analyses suggests that elevated BPV is also associated with cognitive decline and dementia.^7^, ^8^ However, many prior studies were limited by residual confounding, potential reverse causation, and insufficient longitudinal BP measurements, limiting inference regarding independent associations.^9^ Diabetes is often accompanied by autonomic neuropathy, endothelial dysfunction, and insulin resistance, which may amplify BPV by impairing vascular tone regulation, increasing oxidative stress, and enhancing sympathetic activity.^10^, ^11^, ^12^, ^13^ Accordingly, patients with diabetes are generally considered to exhibit higher BPV than non‐diabetic individuals, with levels increasing with longer disease duration and poorer glycemic control.^14^, ^15^ These pathophysiological features raise the possibility that elevated BPV may partly account for the lack of cognitive benefit observed with intensive SBP lowering in populations with diabetes. However, it remains insufficiently characterized whether the cognitive impact of BPV differs by BP treatment intensity in high‐risk populations with and without diabetes, and whether BPV primarily influences the longitudinal rate of cognitive decline rather than only late‐stage categorical cognitive outcomes.

To address these gaps, we conducted a pooled individual‐participant data analysis of SPRINT‐MIND, which enrolled hypertensive patients without diabetes, and the ACCORD‐MIND trial, which enrolled patients with type 2 diabetes. Leveraging the rigorous design of these two randomized controlled trials, this study aimed to examine whether visit‐to‐visit systolic BPV was associated with the longitudinal rate of cognitive decline independent of mean BP, and whether this association varied according to BP treatment intensity across the SPRINT‐MIND and ACCORD‐MIND trial populations.

## 2 METHODS

### 2.1 Study design

We performed a pooled individual participant‐level analysis of two multicenter randomized controlled trials: the SPRINT‐MIND and the ACCORD‐MIND trial. SPRINT‐MIND is a predefined substudy of SPRINT that investigated the effect of intensive SBP lowering to <120 mmHg on the rate of probable dementia and mild cognitive impairment compared with a target of <140 mmHg.^4^ The ACCORD‐MIND study was a substudy of ACCORD trial, a double 2 × 2 factorial parallel‐group randomized clinical trial that tested whether intensive compared with standard management of hyperglycemia, BP, or lipid levels reduced cognitive decline and brain atrophy in patients with type 2 diabetes.^16^ Data were accessed through the National Heart, Lung, and Blood Institute's Biologic Specimen and Data Repository Information Coordinating Centre (BioLINCC; https://biolincc.nhlbi.nih.gov). Details of the trial designs, study populations, interventions, and study procedures have been published previously.^4^, ^5^ Ethics approval for both trials was granted by each participating center. In accordance with institutional policy, further ethics approval was not required for the present analysis (reference PRE.2025.052).

### 2.2 Participants

The majority of participants assigned to intensive BP control required successive modifications to their antihypertensive regimens during the initial 3 months following randomization to achieve target SBP levels.^3^ To ensure reliable calculation of BPV, BP measurements from 3 months post‐randomization onward through the final cognitive assessment were used for BPV quantification. Participants from both the intensive and standard BP treatment groups were included in the present analysis. Subjects were excluded if they met any of the following criteria: (1) no baseline cognitive assessment, as indicated by missing Digit Symbol Substitution Test (DSST) or Digit Symbol Coding Test (DSCT) scores; (2) no cognitive follow‐up, with DSST or DSCT scores unavailable at any subsequent visits; and (3) had fewer than three BP measurements between baseline and follow‐up visit of cognitive assessment.

### 2.3 BP measurements and variability metrics

In both trials, automated oscillometric devices (Omron 907 or 907XL) were used to measure BP at each clinic visit. Participants were seated and three consecutive readings were obtained with their average recorded as the visit BP. In SPRINT, visits occurred monthly during the first 3 months and every 3 months thereafter. In ACCORD trial, visit frequency varied by treatment assignment: participants in intensive glycemia or intensive BP groups were seen monthly in the first 4 months and subsequently every 2 months, whereas those in standard treatment groups were followed approximately every 4 months.

Visit‐to‐visit systolic BPV was calculated using SBP readings from the 3‐month visit through the final available cognitive assessment. The primary metric was variation independent of mean (VIM), given its statistical independence from mean SBP and thus evaluating the variability's unique contribution to cognitive outcomes. The VIM was calculated as 100 × (SD / mean^β), where β was the regression coefficient derived from the natural logarithm of SD regressed on the natural logarithm of mean SBP across all participants. To verify the consistency of our results, three alternative metrics were calculated: (1) standard deviation (SD) of SBP; (2) coefficient of variation (CV), calculated as SD divided by average SBP; and (3) average real variability (ARV), representing the average of the absolute differences between consecutive SBP measurements.

### 2.4 Measurement of cognitive function

Cognitive function was evaluated through comprehensive neuropsychological batteries in both trials, although the specific compositions and assessment schedules varied. In the ACCORD‐MIND study, cognitive assessments were conducted at baseline, 20 months, and 40 months post‐randomization. In SPRINT‐MIND, cognitive assessments were performed at baseline, Year 2, and Year 4 of follow‐up, with additional assessments at study closeout for participants who were more than 1 year beyond their 4‐year visit.

Despite heterogeneity in the overall cognitive batteries, both trials incorporated a closely comparable measure of psychomotor processing speed derived from the Wechsler Adult Intelligence Scale: the DSST (WAIS‐III) in ACCORD‐MIND and the DSCT (WAIS‐IV) in SPRINT‐MIND. These tasks are functionally equivalent, sharing an identical paradigm that requires rapid symbol–number pairing under time constraints, and they demonstrate high construct validity and strong concordance across studies. Processing speed was selected for harmonization because it is particularly sensitive to cerebrovascular pathology and is among the earliest cognitive domains affected by hypertension‐ and diabetes‐related brain injury. In addition, digit symbol–based measures provide a wide, continuous score range with minimal ceiling effects, making them well suited for detecting subtle longitudinal cognitive changes in non‐demented trial populations.

**RESEARCH IN CONTEXT**

1. ***Systematic review***: Visit‐to‐visit blood pressure variability (BPV) is recognized increasingly as a vascular risk factor for cognitive decline, independent of mean blood pressure levels. However, prior studies largely relied on observational designs with limited longitudinal BP measurements, and it remained unclear whether BPV influences the longitudinal rate of cognitive decline and whether this effect is modified by the intensity of BP lowering across diverse cardiometabolic populations.

2. ***Interpretation***: In this pooled analysis of Systolic Blood Pressure Intervention Trial Memory and Cognition in Decreased Hypertension (SPRINT‐MIND) and Action to Control Cardiovascular Risk in Diabetes Memory in Diabetes (ACCORD‐MIND; *n* = 11,104), higher visit‐to‐visit systolic BPV was independently associated with accelerated cognitive decline, irrespective of mean BP. Exploratory analyses suggest that this adverse effect was more pronounced under intensive BP lowering, offering a plausible explanation for the divergent cognitive outcomes of the two trials.

3. ***Future directions***: Future studies should examine whether antihypertensive strategies that preferentially reduce BPV can preserve cognitive function, and whether BPV reduction should be incorporated as a therapeutic target alongside mean BP control in high‐risk populations.

To facilitate cross‐trial comparability and account for inherent differences in the study cohorts, raw scores from each trial were transformed into standardized Z‐scores. These Z‐scores were calculated by subtracting the baseline mean from individual raw scores and dividing the result by the baseline SD. Such approach for processing cognitive test scores has been well accepted among other studies.^17^, ^18^

### 2.5 Outcomes

The primary outcome was the longitudinal change in global cognitive performance over time, quantified as the annual rate of change in standardized DSST/DSCT Z‐score. Cognitive decline was modeled continuously using repeated assessments across follow‐up, with particular interest in the interaction between visit‐to‐visit systolic BPV and time, representing the annual rate of cognitive change.

Prespecified secondary analyses examined potential effect modification by demographic and clinical characteristics. Exploratory analyses were performed to assess potential non‐linear dose–response relationships between BPV and cognitive outcomes. Additional stratified analyses were conducted according to randomized BP treatment intensity in both the SPRINT‐MIND and ACCORD‐MIND trials.

### 2.6 Statistical analysis

Baseline characteristics of the study population were categorized by tertiles of SBP‐VIM. Continuous variables were presented as mean ± SD or the median with the interquartile range (IQR) and compared using one‐way analysis of variance (ANOVA) or the Kruskal‐–Wallis test, respectively. Categorical variables were expressed as frequencies (percentage) and compared using the chi‐square test.

To evaluate the association between BPV and cognitive function, we employed a linear mixed model with the restricted maximum likelihood method. This approach included random intercepts and random slopes for time at participant level to address within‐individual differences and rates of cognitive change. BPV metrics were analysed both as continuous variables (per 10% increment for VIM) and as categorical variables (tertiles). We performed the above analyses using univariate and multivariate models: Model 1 adjusted for age, sex, ethnicity, and education level; Model 2 additionally adjusted for mean SBP; and Model 3 (fully adjusted model), further adjusted for diabetes, hypertension, history of cardiovascular disease, history of stroke, smoking status, body mass index, intensive BP control assignment, depressive symptoms, baseline glucose, low‐density lipoprotein, and statin use. All covariates were included in the models as both fixed main effects and interaction terms with time.

Potential non‐linear relationships were examined to explore the potential dose‐response pattern between BPV metrics and rate of cognitive change, with four knots placed at the 5th, 35th, 65th, and 95th percentiles. Sensitivity analyses were performed by repeating the above analyses using SD, CV, and ARV of SBP. Restricted cubic spline (RCS) models were fully adjusted using the same covariates as Model 3.

Finally, we performed stratified analyses to re‐evaluate association with global cognitive function in different subgroups, according to age group (<70 vs ≥70 years), sex, ethnicity, educational attainment, smoking status, history of diabetes, hypertension, cardiovascular disease, atrial fibrillation, stroke, depressive symptoms, randomization assignment (intensive vs standard BP control), and statin use. To further examine the consistency of findings, we repeated the primary mixed‐effects models separately for SPRINT‐MIND and ACCORD‐MIND cohorts, and within each trial, additionally stratified by randomized BP treatment assignment (intensive vs standard) to assess whether treatment intensity modified the BPV–cognition association within each trial. Potential effect modification was assessed by including three‐way interaction terms between BPV, time, and each subgroup variable in the fully adjusted mixed‐effects models. Because diabetes status was structurally linked to trial cohort, with SPRINT‐MIND excluding participants with diabetes and ACCORD‐MIND enrolling participants with type 2 diabetes, analyses involving diabetes status were interpreted as comparisons between trial contexts rather than as within‐trial tests of diabetes as an effect modifier.

No imputation was implemented considering capability of mixed model in handling data missing at random. All statistical analyses were performed using R software (version 4.5.0), with a two‐sided *p* < 0.05 considered statistically significant.

## 3 RESULTS

### 3.1 Study population and characteristics

**FIGURE 1:**
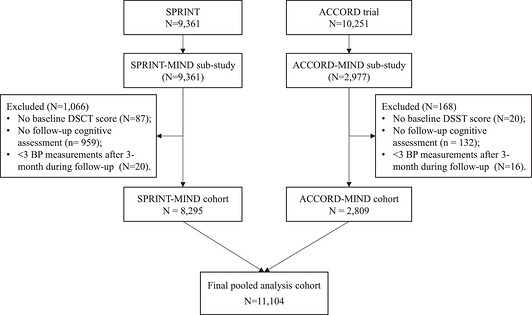
Flowchart of this pooled study. SPRINT‐MIND, Systolic Blood Pressure Intervention Trial Memory and Cognition in Decreased Hypertension; ACCORD‐MIND, Action to Control Cardiovascular Risk in Diabetes Memory in Diabetes; DSCT, Digit Symbol Coding Test; DSST, Digit Symbol Substitution Test; BP, blood pressure.

Of 12,338 participants from the SPRINT‐MIND (n = 9,361) and ACCORD‐MIND (n = 2977) trials, 11,104 were included in the final pooled analysis, with reasons for exclusion shown in Figure 1. During follow‐up, 1132 participants (10.2%) completed one cognitive assessment, 6233 (56.1%) completed two assessments, and 3739 (33.7%) completed three assessments. Characteristics of participants stratified by tertiles of SBP‐VIM are shown in Table 1. The mean age was 66.6 years (SD 8.8), a total of 4212 participants (37.9%) were women, and 6811 (61.3%) were White non‐Hispanic. Participants in the highest SBP‐VIM tertile were older, more likely to be female, and had higher baseline SBP than those in the lowest tertile (all *p*’s < 0.001).

**TABLE 1:** Characteristics of participants according to tertiles of SBP‐VIM.

Variable^*^	Total *N* = 11104	T1 *N* = 3702	T2 *N* = 3701	T3 *N* = 3701
SBP‐VIM	10.5 ± 4.7	5.9 ± 1.7	9.9 ± 1.0	15.7 ± 3.6
Intensive blood pressure control, *n* (%)	4856 (43.7)	1464 (39.5)	1629 (44.0)	1763 (47.6)
Age, years	66.6 ± 8.8	65.5 ± 8.4	66.6 ± 8.6	67.7 ± 9.2
Age ≥70, years, *n* (%)	3887 (35.0)	1099 (29.7)	1274 (34.4)	1514 (40.9)
Female sex, *n* (%)	4212 (37.9)	1156 (31.2)	1375 (37.2)	1681 (45.4)
Ethnicity, *n*. (%)				
White	6811 (61.3)	2288 (61.8)	2313 (62.5)	2210 (59.7)
Black	2907 (26.2)	880 (23.8)	942 (25.5)	1085 (29.3)
Hispanic	1045 (9.4)	407 (11.0)	338 (9.1)	300 (8.1)
Other	341 (3.1)	127 (3.4)	108 (2.9)	106 (2.9)
Education, *n* (%)				
Less than high school graduate	1024 (9.2)	295 (8.0)	334 (9.0)	395 (10.7)
High school graduate or GED	2068 (18.6)	655 (17.7)	663 (17.9)	750 (20.3)
Some college/technical school	3884 (35.0)	1297 (35.0)	1287 (34.8)	1300 (35.1)
College graduate or more	4128 (37.2)	1455 (39.3)	1417 (38.3)	1256 (33.9)
Comorbid conditions, *n* (%)				
Hypertension	9834 (88.6)	3222 (87.0)	3262 (88.1)	3350 (90.5)
Diabetes	2809 (25.3)	965 (26.1)	902 (24.4)	942 (25.5)
Atrial fibrillation	1409 (12.9)	412 (11.3)	465 (12.8)	532 (14.6)
History of cardiovascular disease	2449 (22.1)	731 (19.7)	753 (20.3)	965 (26.1)
History of stroke	178 (1.6)	53 (1.4)	52 (1.4)	73 (2.0)
Depression	2225 (20.1)	716 (19.4)	707 (19.1)	802 (21.7)
Smoking status, *n* (%)				
Never smoked	4975 (44.8)	1704 (46.0)	1681 (45.4)	1590 (43.0)
Former smoker	4792 (43.2)	1592 (43.0)	1594 (43.1)	1606 (43.4)
Current smoker	1329 (12.0)	403 (10.9)	424 (11.5)	502 (13.6)
Body mass index, kg/m^2^	29.86 (26.58–34.03)	29.94 (26.79–33.83)	29.82 (26.69–34.17)	29.85 (26.13–34.23)
Baseline systolic blood pressure, mmHg	138.48 ± 16.16	137.16 ± 14.88	138.29 ± 15.60	139.99 ± 17.74
Baseline diastolic blood pressure, mmHg	77.28 ± 11.65	77.45 ± 11.00	77.50 ± 11.64	76.89 ± 12.26
eGFR, mL/min/1.73 m^2^	75.65 (61.59–90.09)	77.48 (64.92–90.70)	75.90 (62.30–90.10)	72.37 (58.23–89.06)
Baseline serum creatine	0.97 (0.80–1.15)	0.97 (0.80–1.12)	0.96 (0.80–1.14)	0.98 (0.80–1.19)
Baseline total cholesterol, mg/dL	184 (159–213)	184 (159–212)	186 (161–213)	184 (158–214)
Low‐density lipoprotein, mg/dL	107 (85–131)	107 (85–131)	108 (86–131)	105 (84–131)
HDL cholesterol, mg/dL	48 (40–58)	47 (40–56)	48 (40–58)	49 (41–59)
Glucose, mg/L	102.0 (93.0–123.0)	103.0 (94.0–125.0)	102.0 (93.0–120.0)	101.0 (91.0–122.88)
Statin use, *n*o. (%)	5468 (49.5)	1776 (48.2)	1791 (48.7)	1901 (51.8)
Aspirin use, *n*o. (%)	5865 (53.0)	1916 (51.9)	1973 (53.5)	1976 (53.6)
Baseline albumin/creatinine ratio	10.00 (5.88–24.27)	9.00 (5.34‐20.00)	9.68 (5.84–23.42)	11.76 (6.48– 31.00)
Baseline Digit Symbol Substitution/Coding Test, Z score^†^	0.00 ± 1.00	0.09 ± 0.99	0.01 ± 1.01	−0.10 ± 1.00

* Data are presented as mean ± SD, *n* (%), or median (interquartile range). GED, General Educational Development; eGFR, estimated glomerular filtration rate; HDL, high‐density lipoprotein; SBP‐VIM, systolic blood pressure variation independent of mean; T, tertile.. Tertiles of SBP‐VIM were defined as follows: Tertile 1 (lowest, SBP‐VIM <8.15), Tertile 2 (middle, SBP‐VIM 8.15–11.85), and Tertile 3 (highest, SBP‐VIM >11.85).

^†^ The Digit Symbol Substitution Test (DSST) was used in the ACCORD‐MIND trial (score range 0–120), and the Digit Symbol Coding Test (DSCT) was used in the SPRINT‐MIND trial (score range 0–135). Raw scores were transformed into standardized Z‐scores by subtracting the baseline mean and dividing by the baseline SD within each cohort to ensure comparability.

Participants contributed a mean of 12.0 BP measurements (SD 3.3) over a median follow‐up of 3.94 years (IQR 3.34–5.53). Mean SBP and diastolic blood pressure (DBP) during follow‐up were 138.5 mmHg (SD 16.2) and 77.3 mmHg (SD 11.6), with mean systolic BPV metrics of 10.5 (SD 4.7) for VIM, 10.6 mmHg (SD 5.0) for SD, 8.2% (SD 3.7) for CV, and 11.8 mmHg (SD 6.3) for ARV, respectively. The baseline means and SD of raw scores were 51.2 (SD 15.2) for the DSCT in SPRINT‐MIND and 52.8 (SD 15.7) for the DSST in ACCORD‐MIND, which were used to derive cohort‐specific Z‐scores (eTable S1).

### 3.2 Association between SBP‐BPV and cognitive decline

**FIGURE 2:**
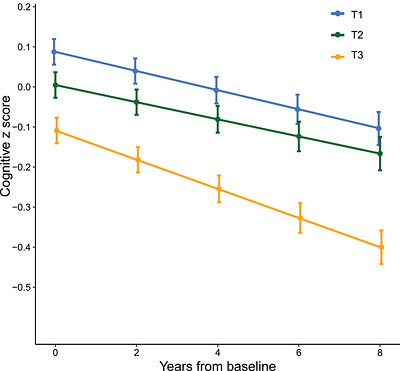
Longitudinal standardized cognitive scores by tertiles of SBP‐VIM. The solid dots represent the average scores, and the error bars represent the 95% confidence intervals. SBP‐VIM, systolic blood pressure variation independent of mean; T1, tertile 1 (lowest tertile, SBP‐VIM: <8.15); T2, tertile 2 (middle tertile, SBP‐VIM: 8.15–11.85); T3, tertile 3 (highest tertile, SBP‐VIM: >11.85).

Longitudinal trajectories of cognitive Z‐scores stratified by SBP‐VIM tertiles are shown in Figure 2 and eFigure S1. Across the pooled cohort, as well as the SPRINT‐MIND and ACCORD‐MIND trials, participants in the highest SBP‐VIM tertile (T3) consistently exhibited a more rapid rate of decline over time compared with those in lower tertiles (T1 and T2). Similar trajectory patterns were observed for SBP‐SD, SBP‐CV, and SBP‐ARV (eFigure S2).

In linear mixed‐effects models, higher SBP‐VIM was independently associated with accelerated cognitive decline (Table 2). When analyzed as a continuous variable, each 10% increment in SBP‐VIM was associated with an additional annual decline of −0.008 Z‐score units (95% confidence interval [CI]: −0.014 to −0.003) in the fully adjusted model, ≈0.12 points per year on the DSCT in SPRINT‐MIND and 0.13 points per year on the DSST in ACCORD‐MIND.

**TABLE 2:** Association between increments in SBP‐VIM and change in standardized cognitive scores.

Parameters	Unadjusted		Model 1		Model 2		Model 3	
	Estimate (95% CI)	*p*	Estimate (95% CI)	*p*	Estimate (95% CI)	*p*	Estimate (95% CI)	*p*
*Continuous variable (per 10% increment)* ^*^								
Time, years	−0.012 (−0.018 to −0.006)	<0.001	0.144 (0.124 to 0.165)	<0.001	0.141 (0.107 to 0.175)	<0.001	0.164 (0.129 to 0.198)	<0.001
SBP‐VIM×time, years	−0.014 (−0.020 to −0.009)	<0.001	−0.010 (−0.015 to −0.004)	<0.001	−0.010 (−0.015 to −0.004)	<0.001	−0.008 (−0.014 to −0.003)	0.003
*Categorical variable (tertiles)*								
Time, years	−0.024 (−0.028 to −0.020)	<0.001	0.136 (0.116 to 0.157)	<0.001	0.131 (0.097 to 0.165)	<0.001	0.157 (0.122 to 0.191)	<0.001
SBP‐VIM T1×time, years	Ref		Ref		Ref		Ref	
SBP‐VIM T2×time, years	0.003 (−0.003 to 0.008)	0.353	0.005 (−0.001 to 0.011)	0.060	0.005 (−0.001 to 0.011)	0.059	0.007 (0.001 to 0.013)	0.015
SBP‐VIM T3×time, years	−0.012 (−0.018 to −0.007)	<0.001	−0.007 (−0.013 to −0.001)	0.018	−0.007 (−0.013 to −0.001)	0.018	−0.005 (−0.012 to −0.001)	0.049

* Coefficients reflected association between 10% (for VIM) increment in BPV and global cognitive Z‐score decline. SBP‐VIM, systolic blood pressure variation independent of mean; DSST, Digit Symbol Substitution Test; DSCT, Digit Symbol Coding Test; T1, tertile 1 (lowest, SBP‐VIM <8.15); T2, tertile 2 (middle, SBP‐VIM 8.15–11.85); T3, tertile 3 (highest, SBP‐VIM >11.85); CI, confidence interval; Ref, reference; BPV, blood pressure variability; SBP, systolic blood pressure.

Model 1: age, sex, ethnicity, and education.

Model 2: age, sex, ethnicity, education, and mean SBP.

Model 3: age, sex, ethnicity, education, and mean SBP, history of cardiovascular disease, stroke, current smoke, diabetes, hypertension, statin use, baseline glucose, low‐density lipoprotein, body mass index, intensive BP control, depression.

In categorical analyses of SBP‐VIM, participants in the highest tertile exhibited a faster rate of decline over follow‐up compared with those in the lowest tertile (Table 2). Specifically, relative to T1, the highest SBP‐VIM tertile was associated with a steeper annual decline (*β* = −0.005 per year; 95% CI: −0.012 to −0.001). Over the median 3.94‐year follow‐up, this corresponded to a cumulative additional decline of ≈0.30 raw score points between the highest and lowest SBP‐VIM tertiles. The interaction between the middle tertile and time was statistically significant (*β* = 0.007 per year; 95% CI: 0.001 to 0.013), indicating a rate of change that differed from the reference group.

When alternative visit‐to‐visit BPV metrics were examined, results were broadly consistent. Higher tertiles of SBP‐SD, SBP‐CV, and SBP‐ARV were each associated with a faster annual decline, whereas the middle tertiles showed no statistically significant association with longitudinal cognitive change in fully adjusted models (eTable S2–S4).

In trial‐specific analyses, the association with the rate of cognitive decline remained statistically significant in the SPRINT‐MIND cohort (β = −0.008 per year; 95% CI: –0.014 to –0.002), whereas it did not reach statistical significance in the overall ACCORD‐MIND cohort (β = −0.014 per year; 95% CI: –0.033 to 0.004) (eTable S5).

### 3.3 Dose–response relationship and non‐linearity

The spline curves demonstrated approximately linear relationships between each BPV metric and the rate of cognitive decline. All four metrics showed statistically significant overall associations with accelerated cognitive decline (*p*‐overall = 0.005 for SBP‐VIM, 0.003 for SBP‐SD, 0.002 for SBP‐CV, and 0.008 for SBP‐ARV). No evidence of non‐linear associations was observed for any BPV metric (*p* for non‐linearity = 0.925 for SBP‐VIM, 0.405 for SBP‐SD, 0.861 for SBP‐CV, and 0.075 for SBP‐ARV; eFigure 3).

### 3.4 Association between BPV and cognitive decline by BP treatment

In the pooled cohort, the adverse association between higher SBP‐VIM and faster cognitive decline appeared more pronounced under intensive than standard control, although the three‐way interaction between SBP‐VIM, treatment assignment, and time was suggestive but did not reach significance (p for interaction = 0.054). When stratified by BP treatment assignment within each trial, higher SBP‐VIM was associated with faster cognitive decline under intensive control (ACCORD‐MIND *β* = −0.035 per year, 95% CI: –0.063 to −0.007; SPRINT‐MIND *β* = −0.013 per year, 95% CI: −0.021 to −0.005) but not under standard control. However, the interaction between SBP‐VIM, BP treatment assignment, and time did not reach statistical significance (*p* for interaction = 0.074 for ACCORD‐MIND and 0.101 for SPRINT‐MIND) (eTables S6–S7).

### 3.5 Subgroup analyses

**FIGURE 3:**
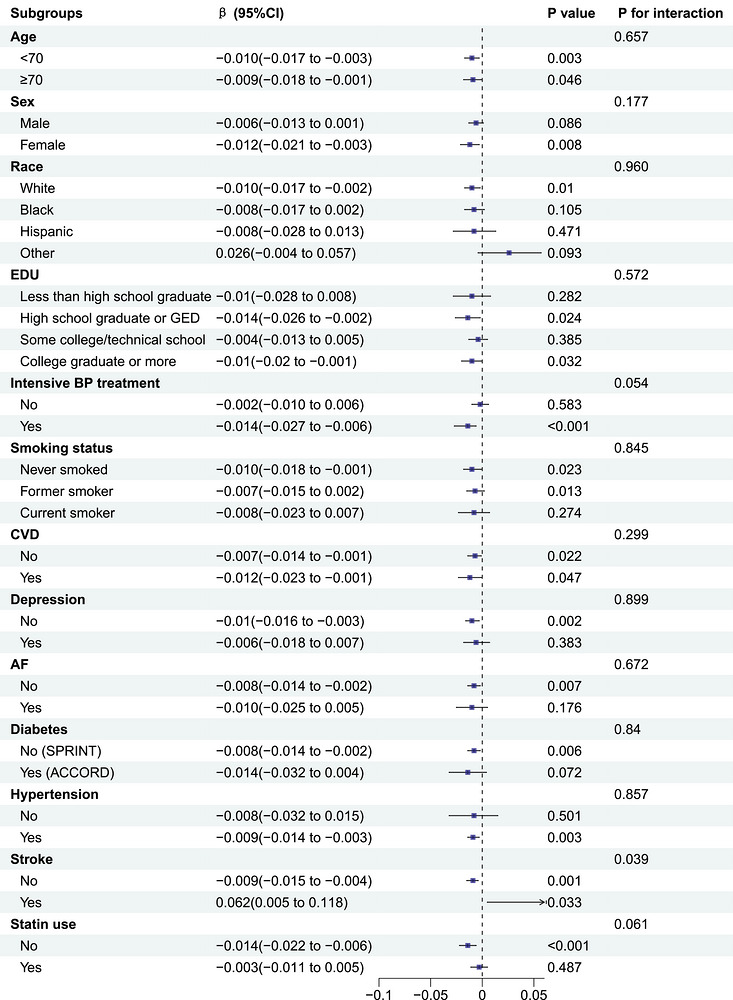
Subgroup analyses of the association of SBP‐VIM with standardized cognitive scores. Implication of coefficients and adjusted covariates were same as results in Model 3. ACCORD‐MIND, Action To Control Cardiovascular Risk In Diabetes Memory In Diabetes; AF, artrial fibrillation; BMI, body mass index; CI, confidence interval; CVD, cardiovascular disease; EDU, educational attainment; GED, General Educational Development; SBP‐VIM, systolic blood pressure variation independent of mean; SPRINT‐MIND, Systolic Blood Pressure Intervention Trial Memory And Cognition In Decreased Hypertension.

In prespecified subgroup analyses (Figure 3), a statistically significant interaction was observed for history of stroke (*p* for interaction = 0.039). The detrimental association between higher SBP‐VIM and faster cognitive decline was stronger in participants without a prior stroke (β = −‐0.009; 95% CI: −0.015 to −0.004). No significant effect modifications were observed for age, sex, ethnicity, diabetes status, cardiovascular disease, atrial fibrillation, statin use, or other covariates (all *p*’s for interaction > 0.05). However, the direction of the association between higher SBP‐VIM and cognitive decline was largely consistent across all subgroups.

## 4 DISCUSSION

In this pooled longitudinal analysis of two large, randomized trial cohorts, we found that higher visit‐to‐visit systolic BPV was independently associated with accelerated cognitive decline over time, even after accounting for mean SBP and a wide range of demographic and clinical covariates. The association was largely linear, observed across multiple BPV metrics, and evident in the pooled cohort. Notably, the adverse association between BPV and cognitive decline appeared more pronounced under intensive BP lowering. Although exploratory, this treatment‐context pattern provides a plausible and testable framework for interpreting the divergent cognitive findings of SPRINT‐MIND and ACCORD‐MIND.

Although the association between BPV and cognitive impairment has been reported previously, the present study extends existing evidence in several important ways. By modeling the continuous rate of decline in psychomotor speed, a cognitive domain particularly sensitive to cerebrovascular disease, rather than relying on late‐stage categorical diagnoses, this study captures early, graded neurocognitive deterioration. In addition, by examining both diabetic and non‐diabetic populations within a unified analytical framework, the present analysis clarifies that the adverse cognitive implications of hemodynamic instability are evident in the pooled cohort and SPRINT‐MIND. These findings suggest that the cognitive impact of BPV may be context dependent, becoming particularly salient under conditions of intensive mean BP lowering. Although exploratory, this observation offers a plausible mechanistic framework for understanding why intensive BP control improved cognitive outcomes in SPRINT‐MIND but not in ACCORD‐MIND. Finally, these findings position BPV as a determinant of the trajectory of cognitive aging that operates independently of mean BP levels, offering a plausible explanation for why intensive SBP lowering does not confer uniform cognitive benefit across individuals.

Our findings reinforce the established consensus that visit‐to‐visit systolic BPV is robustly associated with cognitive decline, independent of absolute BP levels.^8^, ^19^, ^20^, ^21^ Notably, a prior post hoc analysis of SPRINT‐MIND suggested that the detrimental effects of BPV were confined primarily to the standard treatment group.^22^ In contrast, our pooled analysis incorporating the diabetic ACCORD‐MIND population suggests that the adverse impact of BPV is accentuated under intensive BP lowering conditions, a pattern consistent with both the SPRINT‐MIND and ACCORD‐MIND intensive arms. This discrepancy likely arises from our enhanced statistical power, differences in study population, and the use of continuous Z‐score trajectories rather than a categorical outcome. By modeling cognitive change as a continuous process, we captured subtle deteriorations in psychomotor speed that might be obscured by the threshold‐based definitions of cognitive impairment used in earlier reports. Furthermore, with an average of 12 BP measurements per participant, this study may provide a more precise estimation of long‐term BPV than studies relying on fewer assessments, thereby reducing measurement noise and strengthening the observed associations.

The influence of higher BPV on cognitive function likely stems from a cascade of hemodynamic stress and structural cerebral damage. Repeated hemodynamic fluctuations induce endothelial dysfunction, oxidative stress, and arterial stiffening,^23^ which in turn contribute to the development and progression of cerebral small vessel disease, including white matter hyperintensities, lacunar infarcts, microbleeds, and brain atrophy.^24^, ^25^ Such structural damage disrupts cortico‐subcortical connectivity, impairing processing speed, and executive function, the cognitive domains assessed by the DSST/DSCT in this study. In populations with high cardiovascular risk, such as participants in SPRINT and ACCORD, cerebral autoregulation is frequently impaired, reducing the brain's capacity to buffer systemic pressure fluctuations. Persistent hemodynamic instability under these conditions may accelerate cerebral small vessel disease, contributing to cognitive impairment and dementia.^26^, ^27^ Moreover, the relationship between BPV and cognitive decline may be bidirectional or arise from shared underlying pathology, such as cerebral small vessel disease, underscoring the need for further mechanistic investigation.^28^ These findings underscore the potential clinical value of targeting BPV to preserve neural network integrity and slow cognitive deterioration, independent of average BP levels.

The long‐standing discrepancy between SPRINT‐MIND and ACCORD‐MIND raises the possibility that factors beyond mean BP influence cognitive responses to intensive BP lowering. In the present analysis, the BPV‐cognition association was directionally consistent in both the non‐diabetic and diabetic trials; because diabetes status was collinear with trial, the present design cannot determine whether diabetes itself modifies this association. Notably, exploratory stratification within both trials suggested that the adverse cognitive impact of BPV appeared more pronounced among participants receiving intensive BP control, with no such association was observed under standard treatment; however, the treatment‐intensity findings did not reach statistical significance. This pattern may provide a plausible framework for understanding why intensive BP lowering improved cognitive outcomes in SPRINT‐MIND but not in ACCORD‐MIND. In the non‐diabetic SPRINT‐MIND population, where BPV is comparatively lower and more controllable, the mean‐BP benefit may predominate, yielding net cognitive protection; in the diabetic ACCORD‐MIND population, autonomic neuropathy, endothelial dysfunction, and impaired cerebral autoregulation predispose to elevated and treatment‐resistant BPV, so the BPV‐related harm may offset this benefit and produce a null net effect.^29^ In the context of aggressive BP reduction, these underlying vulnerabilities may render the brain particularly susceptible to hemodynamic fluctuations. Even with well‐controlled mean BP, fluctuations in SBP may continue to drive cerebrovascular injury and neurocognitive decline, acting as a residual vascular risk mechanism.^30^ Clinically, these results highlight the importance of complementing mean BP targets with strategies to reduce BPV, through optimized drug selection, timing, or novel interventions, to protect cognitive function. Future trials should test whether targeting BPV alongside conventional risk factor management can preserve cognition in high‐risk populations.

This study has several limitations. First, participants were at high cardiovascular risk, which may limit the generalizability of our findings to lower‐risk populations, and the observational, post hoc nature of the analysis cannot entirely exclude residual confounding or reverse causality. Second, we focused on the DSST/DSCT for longitudinal comparability, assessing primarily processing speed and executive function rather than global cognitive domains, and emphasized continuous cognitive decline rather than categorical outcomes such as incident mild cognitive impairment or dementia; therefore, the clinical significance of these BPV‐related changes remains uncertain. Third, the median follow‐up of 3.94 years may be insufficient to capture the long‐term cumulative effects of BPV or progression to dementia. Fourth, this study did not examine whether different classes of antihypertensive medications modify the BPV–cognition relationship. Fifth, because SPRINT‐MIND excluded participants with diabetes whereas ACCORD‐MIND enrolled participants with type 2 diabetes, diabetes status was structurally linked to trial cohort. Therefore, the present analysis cannot disentangle diabetes‐specific effect modification from other trial‐level differences. Treatment‐intensity analyses should also be interpreted as exploratory. Finally, despite adjustment for a broad range of covariates, residual confounding by factors such as subclinical frailty or genetic predispositions (e.g., apolipoprotein E [*APOE*] ε4) cannot be excluded.

Among adults at elevated cardiovascular risk, higher visit‐to‐visit systolic BPV was independently associated with accelerated cognitive decline after accounting for mean BP. The association was observed in the pooled cohort, with no clear evidence of heterogeneity by diabetes or trial context, although exploratory analyses suggested that BP treatment intensity may influence the strength of the BPV–cognition association. These findings identify BPV as a potential vascular contributor to cerebral end‐organ injury beyond conventional BP control. Prospective studies are needed to determine whether therapeutic strategies that reduce BPV can preserve cognitive function.

## CONFLICT OF INTEREST STATEMENT

The authors declare no conflict of interest. Author disclosures are available in the Supporting Information.

## CONSENT STATEMENT

Informed consent was obtained from all participants in the original Systolic Blood Pressure Intervention Trial Memory and Cognition in Decreased Hypertension (SPRINT) and Action to Control Cardiovascular Risk in Diabetes Memory in Diabetes (ACCORD) trials by the respective enrolling centers. The present study is a secondary analysis of de‐identified, publicly available participant‐level data obtained from the National Heart, Lung, and Blood Institute's Biologic Specimen and Data Repository Information Coordinating Centre (BioLINCC; https://biolincc.nhlbi.nih.gov). In accordance with institutional policy, further ethics approval was not required for the present analysis (reference PRE.2025.052).

## Supporting information



Supporting Information

Supporting Information
