# Supplementary material for: Visit‐to‐visit systolic blood pressure variability, blood pressure treatment intensity, and cognitive decline
Source: Alzheimers Dement. 2026 Jul 31;22(8):e71726. doi: 10.1002/alz.71726 (PMC13426027; doi:10.1002/alz.71726)
Supplement: Supplementary file 1 — Supporting Information [file ALZ-22-e71726-s001.docx]

**Supplemental Materials**

[eFigure 1. Longitudinal Standardized Cognitive Scores by Tertiles of Systolic Blood Pressure Variability (SBP-VIM) 2](#_Toc232547288)

[eFigure 2. Longitudinal Standardized Cognitive Scores by Tertiles of Systolic Blood Pressure Variability Metrics 3](#_Toc232547289)

[eFigure 3. Restricted Cubic Spline Associations Between Systolic Blood Pressure Variability and Annual Cognitive Decline 4](#_Toc232547290)

[eTable 1. Blood pressure metrics and cognitive outcomes in the pooled cohort and in the SPRINT MIND and ACCORD MIND Trials 5](#_Toc232547291)

[eTable 2. Association Between Increments in Systolic Blood Pressure Standard Deviation and Change in Standardized Cognitive Scores 6](#_Toc232547292)

[eTable 3. Association Between Increments in Systolic Blood Pressure Coefficient of Variation and Change in Standardized Cognitive Scores 7](#_Toc232547293)

[eTable 4. Association Between Increments in Systolic Blood Pressure Average Real Variability and Change in Standardized Cognitive Scores 8](#_Toc232547294)

[eTable 5. Association Between Increments in SBP-VIM and Change in Standardized Cognitive Scores in ACCORD-MIND and SPRINT-MIND 9](#_Toc232547295)

[eTable 6. Association Between Increments in SBP-VIM and Change in Standardized DSST Scores Stratified by Blood Pressure Treatment Target in ACCORD-MIND 10](#_Toc232547296)

[eTable 7. Association Between Increment in SBP-VIM and Change in Standardized DSCT scores Stratified by Blood Pressure Treatment Target in SPRINT MIND. 12](#_Toc232547297)

# **eFigure 1. Longitudinal Standardized Cognitive Scores by Tertiles of Systolic Blood Pressure Variability (SBP-VIM)**

The solid dots represent the average scores, and the error bars represent the 95% confidence intervals. SBP-VIM, systolic blood pressure variation independent of mean; T1, tertile 1 (lowest tertile, SBP-VIM: <8.15); T2, tertile 2 (middle tertile, SBP-VIM: 8.15-11.85); T3, tertile 3 (highest tertile, SBP-VIM: >11.85).

# **eFigure 2. Longitudinal Standardized Cognitive Scores by Tertiles of Systolic Blood Pressure Variability Metrics**

The solid dots represent the average scores, and the error bars represent the 95% confidence intervals. SBP-SD, systolic blood pressure standard deviation; SBP-CV, systolic blood pressure coefficient of variation; SBP-ARV, systolic blood pressure average real variability; T1, tertile 1 (lowest tertile); T2, tertile 2 (middle tertile); T3, tertile 3 (highest tertile). Tertile cut points were as follows: SBP-SD (<8.05, 8.05-11.83, >11.83), SBP-CV (<6.38, 6.38-9.25, >9.25), and SBP-ARV (<8.50, 8.50-13.29, >13.29).

# **eFigure 3. Restricted Cubic Spline Associations Between Systolic Blood Pressure Variability and Annual Cognitive Decline**

Restricted cubic spline curves showing dose-response relationships between blood pressure variability metrics and annual cognitive decline rate. Solid lines represent estimated associations, and shaded areas represent 95% confidence intervals. Models were fully adjusted for age, sex, ethnicity, education, mean SBP, cardiovascular disease history, stroke, smoking, diabetes, hypertension, statin use, baseline glucose, LDL, BMI, intensive BP control, and depression.

SBP-VIM, systolic blood pressure variation independent of mean; SBP-SD, standard deviation; SBP-CV, coefficient of variation; SBP-ARV, average real variability.

# **eTable 1.** **Blood pressure metrics and cognitive outcomes in the pooled cohort and in the SPRINT MIND and ACCORD MIND Trials**

| **Variable*** | **Pooled cohort**  **N=11104** | **SPRINT MIND**  **N=8295** | **ACCORD MIND**  **N=2809** |
| --- | --- | --- | --- |
| Baseline Digit Symbol Substitution/Coding Test, mean±SD‡ | - | 51.2±15.2 | 52.8±15.7 |
| Baseline Digit Symbol Substitution/Coding Test, z score‡ | 0.0±1.0 | 0.0±1.0 | 0.0±1.0 |
| No. of BP measurements for BPV calculation | 12.0±3.3 | 12.2±2.9 | 11.6±4.3 |
| Duration of follow up, days, median (IQR) | 1438 (1218-2019) | 1503 (1342-2143) | 1218 (1003-1318) |
| SBP during the follow up period, mean±SD† | 138.5±16.2 | 139.5±15.5 | 135.3±17.6 |
| DBP during the follow up period, mean±SD† | 77.3±11.6 | 78.2±11.8 | 74.7±10.6 |
| Visit-to-visit SBP-VIM, mean±SD† | 10.5±4.7 | 10.5±4.6 | 10.5±4.9 |
| Visit-to-visit SBP-SD, mmHg, mean±SD† | 10.6±5.0 | 10.6±4.9 | 10.6±5.2 |
| Visit-to-visit SBP-CV, %, mean±SD† | 8.2±3.7 | 8.2±3.7 | 8.3±3.9 |
| Visit-to-visit SBP-ARV, mmHg, mean±SD† | 11.8±6.3 | 12.0±6.7 | 11.8±6.1 |

*Data are presented as mean±SD, n (%), or median (interquartile range). BP, blood pressure; BPV, blood pressure variability; DBP, diastolic blood pressure; DSCT, Digit Symbol Coding Test; DSST, Digit Symbol Substitution Test; IQR, interquartile range; SBP, systolic blood pressure; SD, standard deviation.

†Values are calculated based on the included blood pressure measurement included, i.e., all BP measurements starting at the 3 months until the occurrence of a major cardiovascular events or trial completion.

‡The Digit Symbol Substitution Test (DSST, score range 0-120) was administered in ACCORD-MIND, while the Digit Symbol Coding Test (DSCT, score range 0-135) was used in SPRINT-MIND. Raw scores were standardized into z-scores by subtracting the trial-specific baseline mean and dividing by the baseline standard deviation to ensure comparability across cohorts.

# **eTable 2. Association Between Increments in Systolic Blood Pressure Standard Deviation and Change in Standardized Cognitive Scores**

|  | **Unadjusted** | | **Model 1** | | **Model 2** | | **Model 3** | |
| --- | --- | --- | --- | --- | --- | --- | --- | --- |
|  | **Estimate (95% CI)** | **P** | **Estimate (95% CI)** | **P** | **Estimate (95% CI)** | **P** | **Estimate (95% CI)** | **P** |
| *Continuous variable (per 10 mmHg increment), SBP-SD** | | | | | | | | |
| Time (years) | -0.013 (-0.018, -0.007) | <0.001 | 0.143 (0.123, 0.164) | <0.001 | 0.127 (0.093, 0.161) | <0.001 | 0.164 (0.129, 0.168) | <0.001 |
| SBP-SD | -0.230 (-0.267, -0.193) | <0.001 | -0.114 (-0.145, -0.082) | <0.001 | -0.109 (-0.142, -0.077) | <0.001 | -0.080 (-0.117, -0.044) | <0.001 |
| SBP-SD×time (years) | -0.014 (-0.019, -0.009) | <0.001 | -0.009 (-0.013, -0.004) | <0.001 | -0.009 (-0.014, -0.004) | <0.001 | -0.008 (-0.013, -0.002) | 0.005 |
| *Categorical variable (tertiles), SBP-SD* | | | | | | | | |
| Time (years) | -0.024 (-0.028, -0.020) | <0.001 | 0.136 (0.116, 0.157) | <0.001 | 0.131 (0.097, 0.165) | <0.001 | 0.157 (0.123, 0.192) | <0.001 |
| SBP-SD T1 | Ref |  | Ref |  | Ref |  | Ref |  |
| SBP-SD T2 | -0.083 (-0.128, -0.038) | 0.001 | -0.047 (-0.085, -0.009) | 0.036 | -0.048 (-0.085, -0.010) | 0.013 | -0.044 (-0.085, -0.003) | 0.036 |
| SBP-SD T3 | -0.196 (-0.241, -0.151) | <0.001 | -0.087 (-0.125, -0.049) | <0.001 | -0.086 (-0.125, -0.048) | <0.001 | -0.076 (-0.119, -0.033) | <0.001 |
| SBP-SD T1×time (years) | Ref |  | Ref |  | Ref |  | Ref |  |
| SBP-SD T2×time (years) | 0.003 (-0.003, 0.008) | 0.353 | 0.005 (-0.001, 0.011) | 0.060 | 0.005 (-0.001, 0.011) | 0.059 | 0.004 (-0.002, 0.010) | 0.154 |
| SBP-SD T3×time (years) | -0.012 (-0.018, -0.007) | <0.001 | -0.007 (-0.013, -0.001) | 0.018 | -0.007 (-0.013, -0.001) | 0.018 | -0.007 (-0.013, -0.002) | 0.030 |

SBP-SD, systolic blood pressure standard deviation; DSST, Digit Symbol Substitution Test; DSCT, Digit Symbol Coding Test; T1, tertile 1 (lowest); T2, tertile 2; T3, tertile 3 (highest); CI, confidence interval; Ref, reference; BPV, blood pressure variability; SBP, systolic blood pressure. Tertiles of SBP-SD were defined as follows: Tertile 1 (lowest, SBP-SD <8.05), Tertile 2 (middle, SBP-SD 8.05-11.83), and Tertile 3 (highest, SBP-SD >11.83)

*Coefficients reflected association between per 10 mmHg (for SD) increment in BPV and global cognitive Z−score decline.

Model 1: age, sex, ethnicity, and education;

Model 2: age, sex, ethnicity, education, and mean SBP (n=10919)

Model 3: age, sex, ethnicity, education, and mean SBP, history of cardiovascular disease, stroke, current smoke, diabetes, hypertension, statin use, baseline glucose, low-density lipoprotein, body mass index, intensive BP control, depression. (n=10882).

# **eTable 3. Association Between Increments in Systolic Blood Pressure Coefficient of Variation and Change in Standardized Cognitive Scores**

|  | **Unadjusted** | | **Model 1** | | **Model 2** | | **Model 3** | |
| --- | --- | --- | --- | --- | --- | --- | --- | --- |
|  | **Estimate (95% CI)** | **P** | **Estimate (95% CI)** | **P** | **Estimate (95% CI)** | **P** | **Estimate (95% CI)** | **P** |
| *Continuous variable (per 10% increment), SBP-CV** | | | | | | | | |
| Time (years) | -0.012 (-0.018,-0.006) | <0.001 | 0.144 (0.124, 0.165) | <0.001 | 0.137 (0.104, 0.171) | <0.001 | 0.164 (0.129, 0.198) | <0.001 |
| SBP-CV | -0.279 (-0.329, -0.229) | <0.001 | -0.146 (-0.188, -0.103) | <0.001 | -0.142 (-0.184, -0.099) | <0.001 | -0.103 (-0.150, -0.056) | <0.001 |
| SBP-CV×time (years) | -0.018 (-0.025,-0.012) | <0.001 | -0.012 (-0.019, -0.006) | <0.001 | -0.012 (-0.019, -0.006) | <0.001 | -0.011 (-0.018, -0.004) | 0.003 |
| *Categorical variable (tertiles), SBP-CV* | | | | | | | | |
| Time (years) | -0.024 (-0.028, -0.020) | <0.001 | 0.135 (0.115, 0.156) | <0.001 | 0.129 (0.095, 0.163) | <0.001 | 0.157 (0.112, 0.191) | <0.001 |
| SBP-CV T1 | Ref |  | Ref |  | Ref |  | Ref |  |
| SBP-CV T2 | -0.091 (-0.136, -0.046) | <0.001 | -0.048 (-0.086, -0.010) | 0.012 | -0.047 (-0.085, -0.010) | 0.014 | -0.044 (-0.085, -0.003) | 0.035 |
| SBP-CV T3 | -0.207 (-0.252, -0.161) | <0.001 | -0.096 (-0.134, -0.058) | <0.001 | -0.093 (-0.131, -0.055) | <0.001 | -0.073 (-0.115, -0.031) | <0.001 |
| SBP-CV T1×time (years) | Ref |  | Ref |  | Ref |  | Ref |  |
| SBP-CV T2×time (years) | 0.003 (-0.002, 0.009) | 0.265 | 0.006 (0.001, 0.012) | 0.028 | 0.006 (0.001, 0.012) | 0.029 | 0.009 (0.003, 0.015) | 0.005 |
| SBP-CV T3×time (years) | -0.012 (-0.018, -0.006) | <0.001 | -0.006 (-0.012, -0.001) | 0.035 | -0.006 (-0.012, -0.001) | 0.033 | -0.005 (-0.011, 0.002) | 0.116 |

SBP-SD, systolic blood pressure standard deviation; DSST, Digit Symbol Substitution Test; DSCT, Digit Symbol Coding Test; T1, tertile 1 (lowest); T2, tertile 2; T3, tertile 3 (highest); CI, confidence interval; Ref, reference; BPV, blood pressure variability; SBP, systolic blood pressure. Tertiles of SBP-CV were defined as follows: Tertile 1 (lowest, SBP-CV <6.38), Tertile 2 (middle, SBP-CV 6.38-9.25), and Tertile 3 (highest, SBP-CV >9.25)

*Coefficients reflected association between per 10% for CV increment in BPV and global cognitive Z−score decline.

Model 1: age, sex, ethnicity, and education;

Model 2: age, sex, ethnicity, education, and mean SBP (n=10919)

Model 3: age, sex, ethnicity, education, and mean SBP, history of cardiovascular disease, stroke, current smoke, diabetes, hypertension, statin use, baseline glucose, low-density lipoprotein, body mass index, intensive BP control, depression. (n=10882).

# **eTable 4. Association Between Increments in Systolic Blood Pressure Average Real Variability and Change in Standardized Cognitive Scores**

|  | **Unadjusted** | | **Model 1** | | | **Model 2** | | | **Model 3** | |
| --- | --- | --- | --- | --- | --- | --- | --- | --- | --- | --- |
|  | **Estimate (95% CI)** | **P** | **Estimate (95% CI)** | **P** | **Estimate (95% CI)** | | **P** | **Estimate (95% CI)** | | **P** |
| *Continuous variable (per 10% increment), SBP-ARV** | | | | | | | | | | |
| Time (years) | -0.017 (-0.022,-0.011) | <0.001 | 0.142 (0.121, 0.162) | <0.001 | 0.129 (0.095, 0.163) | | <0.001 | 0.162 (0.128, 0.197) | | <0.001 |
| SBP-ARV | -0.175 (-0.205, -0.146) | <0.001 | -0.089 (-0.114, -0.064) | <0.001 | -0.085 (-0.111, -0.059) | | <0.001 | -0.064 (-0.092, -0.035) | | <0.001 |
| SBP-ARV×time (years) | -0.009 (-0.013,-0.005) | <0.001 | -0.005 (-0.009, -0.001) | 0.008 | -0.006 (-0.010, -0.002) | | 0.005 | -0.004 (-0.008, -0.001) | | 0.022 |
| *Categorical variable (tertiles), SBP-ARV* | | | | | | | | | | |
| Time (years) | -0.021 (-0.025, -0.017) | <0.001 | 0.138 (0.118, 0.059) | <0.001 | 0.128 (0.094, 0.162) | | <0.001 | 0.161 (0.126, 0.195) | | <0.001 |
| SBP-ARV T1 | Ref |  | Ref |  | Ref | |  | Ref | |  |
| SBP-ARV T2 | -0.064 (-0.109, -0.019) | 0.005 | -0.023 (-0.061, 0.015) | 0.233 | -0.021 (-0.058, 0.017) | | 0.285 | -0.011 (-0.052, 0.030) | | 0.604 |
| SBP-ARV T3 | -0.240 (-0.285, -0.195) | <0.001 | -0.122 (-0.161, -0.084) | <0.001 | -0.116 (-0.155, -0.077) | | <0.001 | -0.092 (-0.135, -0.049) | | <0.001 |
| SBP-ARV T1×time (years) | Ref |  | Ref |  | Ref | |  | Ref | |  |
| SBP-ARV T2×time (years) | -0.006 (-0.011, 0.001) | 0.051 | 0.002 (-0.008, 0.004) | 0.463 | 0.002 (-0.008, 0.003) | | 0.428 | -0.002 (-0.008, 0.004) | | 0.523 |
| SBP-ARV T3×time (years) | -0.013 (-0.018, -0.007) | <0.001 | -0.007 (-0.013, -0.001) | 0.023 | -0.007 (-0.013, -0.001) | | 0.017 | -0.005 (-0.011, -0.002) | | 0.048 |

SBP-SD, systolic blood pressure standard deviation; DSST, Digit Symbol Substitution Test; DSCT, Digit Symbol Coding Test; T1, tertile 1 (lowest); T2, tertile 2; T3, tertile 3 (highest); CI, confidence interval; Ref, reference; BPV, blood pressure variability; SBP, systolic blood pressure. Tertiles of SBP-ARV were defined as follows: Tertile 1 (lowest, SBP-ARV <8.50), Tertile 2 (middle, SBP-ARV 8.50-13.29), and Tertile 3 (highest, SBP-ARV >13.29)

*Coefficients reflected association between per 10% for ARV increment in BPV and global cognitive Z−score decline.

Model 1: age, sex, ethnicity, and education;

Model 2: age, sex, ethnicity, education, and mean SBP (n=10919)

Model 3: age, sex, ethnicity, education, and mean SBP, history of cardiovascular disease, stroke, current smoke, diabetes, hypertension, statin use, baseline glucose, low-density lipoprotein, body mass index, intensive BP control, depression. (n=10882).

# **eTable 5. Association Between Increments in SBP-VIM and Change in Standardized Cognitive Scores in ACCORD-MIND and SPRINT-MIND**

|  | **Unadjusted** | | | **Model 1** | | | **Model 2** | | | **Model 3** | |
| --- | --- | --- | --- | --- | --- | --- | --- | --- | --- | --- | --- |
|  | **Estimate (95% CI)** | **P** | **Estimate (95% CI)** | | **P** | **Estimate (95% CI)** | | **P** | **Estimate (95% CI)** | | **P** |
| **ACCORD MIND** | | | | | | | | | | | |
| *Continuous variable (per 10% increment)** | | | | | | | | | | | |
| Time (years) | -0.023 (-0.037, -0.009) | <0.001 | 0.087 (0.019, 0.155) | | 0.013 | 0.155 (0.068, 0.243) | | <0.001 | 0.167 (0.040, 0.293) | | 0.010 |
| SBP-VIM | -0.213 (-0.288, -0.138) | <0.001 | -0.134 (-0.194, -0.074) | | <0.001 | -0.132 (-0.192, -0.072) | | <0.001 | -0.125 (-0.218, -0.032) | | 0.009 |
| SBP-VIM×time (years) | -0.011 (-0.023, 0.001) | 0.078 | -0.012 (-0.024, 0.001) | | 0.052 | -0.012 (-0.024, 0.001) | | 0.061 | -0.014 (-0.033, 0.004) | | 0.072 |
| *Categorical variable (tertiles)* | | | | | | | | | | | |
| Time (years) | -0.020 (-0.034, -0.006) | 0.004 | 0.081 (0.014, 0.149) | | 0.018 | 0.152 (0.064, 0.239) | | <0.001 | 0.162 (0.036, 0.289) | | 0.012 |
| SBP-VIM T1 | Ref |  | Ref | |  | Ref | |  | Ref | |  |
| SBP-VIM T2 | -0.047 (-0.136, 0.043) | 0.309 | -0.009 (-0.023, 0.005) | | 0.207 | -0.021 (-0.092, 0.050) | | 0.560 | 0.020 (-0.086, 0.126) | | 0.713 |
| SBP-VIM T3 | -0.188 (-0.277, -0.099) | <0.001 | -0.014 (-0.028, 0.001) | | 0.055 | -0.097 (-0.168, -0.026) | | 0.007 | -0.101 (-0.206, 0.004) | | 0.060 |
| SBP-VIM T1×time (years) | Ref |  | Ref | |  | Ref | |  | Ref | |  |
| SBP-VIM T2×time (years) | -0.009 (-0.023, 0.005) | 0.215 | 0.005 (-0.001, 0.011) | | 0.060 | -0.010 (-0.024, 0.005) | | 0.182 | -0.012 (-0.033, 0.008) | | 0.246 |
| SBP-VIM T3×time (years) | -0.013 (-0.027, 0.001) | 0.079 | -0.007 (-0.013, -0.001) | | 0.018 | -0.013 (-0.028, 0.001) | | 0.061 | -0.017 (-0.038, 0.003) | | 0.098 |
| **SPRINT MIND** | | | | | | | | | | | |
| *Continuous variable (per 10% increment)** | | | | | | | | | | | |
| Time (years) | -0.015 (-0.021, -0.009) | <0.001 | 0.164 (0.142, 0.187) | | <0.001 | 0.144 (0.106, 0.182) | | <0.001 | 0.170 (0.131, 0.208) | | <0.001 |
| SBP-VIM | -0.211 (-0.257, -0.165) | <0.001 | -0.097 (-0.136, -0.058) | | <0.001 | -0.097 (-0.136, -0.057) | | <0.001 | -0.071 (-0.111, -0.030) | | <0.001 |
| SBP-VIM×time (years) | -0.010 (-0.017, -0.004) | 0.002 | -0.009 (-0.014, -0.003) | | 0.002 | -0.009 (-0.015, -0.003) | | 0.002 | -0.008 (-0.014, -0.002) | | 0.006 |
| *Categorical variable (tertiles)* | | | | | | | | | | | |
| Time (years) | -0.023 (-0.028, -0.019) | <0.001 | 0.155 (0.133, 0.178) | | <0.001 | 0.134 (0.096, 0.172) | | <0.001 | 0.163 (0.124, 0.201) | | <0.001 |
| SBP-VIM T1 | Ref |  | Ref | |  | Ref | |  | Ref | |  |
| SBP-VIM T2 | -0.093 (-0.145, -0.041) | <0.001 | -0.054 (-0.098, -0.010) | | 0.015 | -0.055 (-0.099, -0.011) | | 0.015 | -0.053 (-0.097, -0.008) | | 0.020 |
| SBP-VIM T3 | -0.199 (-0.252, -0.147) | <0.001 | -0.077 (-0.122, -0.032) | | <0.001 | -0.077 (-0.122, -0.032) | | <0.001 | -0.056 (-0.101, -0.010) | | 0.017 |
| SBP-VIM T1×time (years) | Ref |  | Ref | |  | Ref | |  | Ref | |  |
| SBP-VIM T2×time (years) | 0.004 (-0.002, 0.011) | 0.174 | 0.008 (0.002, 0.014) | | 0.013 | 0.008 (0.002, 0.014) | | 0.012 | 0.009 (0.003, 0.015) | | 0.005 |
| SBP-VIM T3×time (years) | -0.012 (-0.019, -0.006) | <0.001 | -0.005 (-0.012, 0.001) | | 0.093 | -0.005 (-0.012, 0.001) | | 0.090 | -0.005 (-0.011, 0.002) | | 0.165 |

SBP-VIM, systolic blood pressure variation independent of mean; DSST, Digit Symbol Substitution Test; DSCT, Digit Symbol Coding Test; T1, tertile 1 (lowest); T2, tertile 2; T3, tertile 3 (highest); CI, confidence interval; Ref, reference; BPV, blood pressure variability; SBP, systolic blood pressure. In ACCORD-MIND, fully adjusted (Model 3) analyses were restricted to participants with a randomized BP-treatment assignment in the ACCORD BP trial.

*Coefficients reflected association between 10% (for VIM) increment in BPV and global cognitive Z−score decline.

Model 1: age, sex, ethnicity, and education;

Model 2: age, sex, ethnicity, education, and mean SBP(ACCORD MIND: n=2,809; SPRINT MIND: n=8,295);

Model 3: age, sex, ethnicity, education, and mean SBP, history of cardiovascular disease, stroke, current smoke, hypertension, statin use, baseline glucose, low-density lipoprotein, body mass index, intensive BP control, depression. (ACCORD MIND: n=1,350; SPRINT MIND: n=8,095).

# **eTable 6. Association Between Increments in SBP-VIM and Change in Standardized DSST Scores Stratified by Blood Pressure Treatment Target in ACCORD-MIND**

|  | **Unadjusted** | | **Model 1** | | | **Model 2** | | **Model 3** | |
| --- | --- | --- | --- | --- | --- | --- | --- | --- | --- |
|  | **Estimate (95% CI)** | **P** | **Estimate (95% CI)** | **P** | **Estimate (95% CI)** | | **P** | **Estimate (95% CI)** | **P** |
| **Standard BP Control†** | | | | | | | | | |
| *Continuous variable (per 10% increment)** | | | | | | | | | |
| Time (years) | -0.035 (-0.062, -0.007) | 0.013 | 0.022 (-0.114, 0.157) | 0.754 | 0.129 (-0.080, 0.339) | | 0.226 | 0.105 (-0.075, 0.285) | 0.253 |
| SBP-VIM | -0.165 (-0.319, -0.012) | 0.035 | -0.099 (-0.224, 0.025) | 0.118 | -0.099 (-0.224, 0.025) | | 0.118 | -0.083 (-0.208, 0.043) | 0.196 |
| SBP-VIM×time (years) | 0.003 (-0.022, 0.028) | 0.804 | 0.001 (-0.024, 0.027) | 0.929 | 0.002 (-0.024, 0.027) | | 0.891 | 0.002 (-0.024, 0.027) | 0.884 |
| *Categorical variable (tertiles)* | | | | | | | | | |
| Time (years) | -0.027 (-0.046, -0.008) | 0.005 | 0.028 (-0.106, 0.163) | 0.681 | 0.138 (-0.071, 0.347) | | 0.196 | 0.112 (-0.067, 0.292) | 0.220 |
| SBP-VIM T1 | Ref |  | Ref |  | Ref | |  | Ref |  |
| SBP-VIM T2 | 0.097 (-0.087, 0.282) | 0.301 | 0.017 (-0.131, 0.165) | 0.820 | 0.018 (-0.130, 0.165) | | 0.813 | 0.041 (-0.107, 0.190) | 0.585 |
| SBP-VIM T3 | -0.134 (-0.317, 0.048) | 0.150 | -0.070 (-0.218, 0.078) | 0.352 | -0.069 (-0.217, 0.079) | | 0.359 | -0.047 (-0.197, 0.102) | 0.535 |
| SBP-VIM T1×time (years) | Ref |  | Ref |  | Ref | |  | Ref |  |
| SBP-VIM T2×time (years) | -0.014 (-0.043, 0.015) | 0.337 | -0.014 (-0.043, 0.015) | 0.347 | -0.014 (-0.043, 0.015) | | 0.350 | -0.012 (-0.042, 0.017) | 0.411 |
| SBP-VIM T3×time (years) | -0.001 (-0.029, 0.029) | 0.990 | -0.002 (-0.031, 0.028) | 0.906 | -0.001 (-0.030, 0.029) | | 0.991 | -0.003 (-0.032, 0.027) | 0.858 |
| **Intensive BP Control** | | | | | | | | | |
| *Continuous variable (per 10% increment)** | | | | | | | | | |
| Time (years) | -0.001 (-0.032, 0.032) | 0.992 | 0.277 (0.139, 0.414) | <0.001 | 0.406 (0.216, 0.597) | | <0.001 | 0.233 (0.053, 0.414) | 0.011 |
| SBP-VIM | -0.362 (-0.539, -0.186) | <0.001 | -0.206 (-0.346, -0.067) | 0.004 | -0.202 (-0.343, -0.062) | | 0.005 | -0.106 (-0.168, -0.045) | <0.001 |
| SBP-VIM×time (years) | -0.034 (-0.061, -0.007) | 0.015 | -0.034 (-0.061, -0.006) | 0.015 | -0.030 (-0.057, -0.003) | | 0.032 | -0.035 (-0.063, -0.007) | 0.013 |
| *Categorical variable (tertiles)* | | | | | | | | | |
| Time (years) | -0.022 (-0.044, 0.001) | 0.053 | 0.263 (0.126, 0.400) | <0.001 | 0.399 (0.207, 0.590) | | <0.001 | 0.218 (0.036, 0.399) | 0.019 |
| SBP-VIM T1 | Ref |  | Ref |  | Ref | |  | Ref |  |
| SBP-VIM T2 | -0.041 (-0.238, 0.156) | 0.684 | 0.005 (-0.149, 0.159) | 0.948 | 0.005 (-0.148, 0.159) | | 0.945 | -0.013 (-0.165, 0.140) | 0.872 |
| SBP-VIM T3 | -0.297 (-0.489, -0.105) | 0.003 | -0.177 (-0.327, -0.026) | 0.022 | -0.173 (-0.325, -0.022) | | 0.025 | -0.149 (-0.300, 0.002) | 0.053 |
| SBP-VIM T1×time (years) | Ref |  | Ref |  | Ref | |  | Ref |  |
| SBP-VIM T2×time (years) | -0.012 (-0.041, 0.017) | 0.418 | -0.014 (-0.043, 0.015) | 0.346 | -0.013 (-0.042, 0.015) | | 0.357 | -0.013 (-0.043, 0.016) | 0.367 |
| SBP-VIM T3×time (years) | -0.029 (-0.058, -0.001) | 0.046 | -0.031 (-0.059, -0.002) | 0.035 | -0.027 (-0.056, 0.001) | | 0.062 | -0.032 (-0.061, -0.003) | 0.030 |

SBP-VIM, systolic blood pressure variation independent of mean; DSST, Digit Symbol Substitution Test; DSCT, Digit Symbol Coding Test; T1, tertile 1 (lowest); T2, tertile 2; T3, tertile 3 (highest); CI, confidence interval; Ref, reference; BPV, blood pressure variability; SBP, systolic blood pressure. In ACCORD-MIND, fully adjusted (Model 3) analyses were restricted to participants with a randomized BP-treatment assignment in the ACCORD BP trial.

*Coefficients reflected association between 10% (for VIM) increment in BPV and global cognitive Z−score decline.

†Participants were stratified according to their randomized blood pressure treatment assignment in the ACCORD trial: intensive treatment (systolic blood pressure target <120 mmHg) versus standard treatment (systolic blood pressure target <140 mmHg). P for the SBP-VIM × treatment assignment × time interaction within ACCORD-MIND = 0.074.

Model 1: age, sex, ethnicity, and education;

Model 2: age, sex, ethnicity, education, and mean SBP (Standard BP control: n=663; Intensive BP control: n=695);

Model 3: age, sex, ethnicity, education, and mean SBP, history of cardiovascular disease, stroke, current smoke, hypertension, statin use, baseline glucose, low-density lipoprotein, body mass index, depression (Standard BP control: n=657; Intensive BP control: n=693).

# **eTable 7. Association Between Increment in SBP-VIM and Change in Standardized DSCT scores Stratified by Blood Pressure Treatment Target in SPRINT MIND.**

| BPV parameters | Unadjusted | | Model 1 | | Model 2 | | Model 3 | |
| --- | --- | --- | --- | --- | --- | --- | --- | --- |
|  | Estimate  (95% CI) | P | Estimate  (95% CI) | P | Estimate  (95% CI) | P | Estimate  (95% CI) | P |
| **Standard BP Control†** | | | | | | | | |
| *Continuous variable (per 10% increment)** |  |  |  |  |  |  |  |  |
| Time (years) | -0.013 (-0.022, -0.004) | 0.006 | 0.183 (0.151, 0.215) | <0.001 | 0.135 (0.059, 0.210) | <0.001 | 0.184 (0.129, 0.239) | <0.001 |
| SBP-VIM | -0.214 (-0.283, -0.145) | <0.001 | -0.118 (-0.178, -0.059) | <0.001 | -0.119 (-0.179, -0.059) | <0.001 | -0.100 (-0.161, -0.039) | 0.001 |
| SBP-VIM×time (years) | -0.011 (-0.020, -0.003) | 0.009 | -0.004 (-0.013, 0.004) | 0.325 | -0.004 (-0.012, 0.005) | 0.361 | -0.002 (-0.011, 0.006) | 0.609 |
| *Categorical variable (tertiles)* |  |  |  |  |  |  |  |  |
| Time (years) | -0.022 (-0.028, -0.016) | <0.001 | 0.177 (0.145, 0.209) | <0.001 | 0.127 (0.052, 0.203) | <0.001 | 0.179 (0.124, 0.233) | <0.001 |
| SBP-VIM T1 | Ref |  | Ref |  | Ref |  | Ref |  |
| SBP-VIM T2 | -0.086 (-0.159, -0.012) | 0.022 | -0.059 (-0.121, 0.003) | 0.064 | -0.059 (-0.122, 0.003) | 0.062 | -0.063 (-0.127, -0.001) | 0.049 |
| SBP-VIM T3 | -0.179 (-0.255, -0.104) | <0.001 | -0.084 (-0.149, -0.019) | 0.011 | -0.085 (-0.150, -0.020) | 0.011 | -0.074 (-0.140, -0.008) | 0.029 |
| SBP-VIM T1×time (years) | Ref |  | Ref |  | Ref |  | Ref |  |
| SBP-VIM T2×time (years) | 0.004 (-0.005, 0.012) | 0.430 | 0.006 (-0.002, 0.015) | 0.141 | 0.007 (-0.002, 0.015) | 0.128 | 0.009 (0.001, 0.018) | 0.034 |
| SBP-VIM T3×time (years) | -0.012 (-0.021, -0.003) | 0.010 | -0.005 (-0.014, 0.004) | 0.244 | -0.005 (-0.014, 0.004) | 0.266 | -0.003 (-0.012, 0.006) | 0.486 |
| **Intensive BP Control** | | | | | | | | |
| *Continuous variable (per 10% increment)** |  |  |  |  |  |  |  |  |
| Time (years) | -0.008 (-0.017, 0.001) | 0.062 | 0.145 (0.113, 0.177) | <0.001 | 0.147 (0.085, 0.210) | <0.001 | 0.152 (0.098, 0.206) | <0.001 |
| SBP-VIM | -0.209 (-0.271, -0.147) | <0.001 | -0.075 (-0.128, -0.023) | 0.005 | -0.061 (-0.114,-0.007) | 0.026 | -0.038 (-0.092, 0.016) | 0.171 |
| SBP-VIM×time (years) | -0.018 (-0.025, -0.010) | <0.001 | -0.012 (-0.020, -0.005) | 0.002 | -0.012 (-0.020, -0.004) | 0.012 | -0.013 (-0.021, -0.005) | 0.001 |
| *Categorical variable (tertiles)* |  |  |  |  |  |  |  |  |
| Time (years) | -0.025 (-0.031, -0.019) | <0.001 | 0.133 (0.102, 0.165) | <0.001 | 0.144 (0.081, 0.206) | <0.001 | 0.143 (0.089, 0.197) | <0.001 |
| SBP-VIM T1 | Ref |  | Ref |  | Ref |  | Ref |  |
| SBP-VIM T2 | -0.102 (-0.177, -0.028) | 0.007 | -0.047 (-0.110, 0.015) | 0.136 | -0.042 (-0.104, 0.021) | 0.192 | -0.036 (-0.099, 0.027) | 0.259 |
| SBP-VIM T3 | -0.219 (-0.292, -0.146) | <0.001 | -0.065 (-0.128, -0.003) | 0.041 | -0.049 (-0.113, 0.014) | 0.126 | -0.029 (-0.093, 0.036) | 0.386 |
| SBP-VIM T1×time (years) | Ref |  | Ref |  | Ref |  | Ref |  |
| SBP-VIM T2×time (years) | 0.005 (-0.004, 0.014) | 0.234 | 0.009 (0.001, 0.018) | 0.037 | 0.009 (0.001, 0.018) | 0.036 | 0.009 (-0.001, 0.018) | 0.057 |
| SBP-VIM T3×time (years) | -0.012 (-0.021, -0.004) | 0.006 | -0.005 (-0.014, 0.004) | 0.267 | -0.005 (-0.014, 0.004) | 0.299 | -0.006 (-0.015, 0.003) | 0.200 |

*Coefficients reflected association between 10% (for VIM) increment in BPV and global cognitive Z−score decline.

†Participants were stratified according to their randomized blood pressure treatment assignment in the SPRINT trial: intensive treatment (systolic blood pressure target <120 mmHg) versus standard treatment (systolic blood pressure target <140 mmHg). P for the SBP-VIM × treatment assignment × time interaction within SPRINT-MIND = 0.101.

Model 1: age, sex, ethnicity, and education;

Model 2: age, sex, ethnicity, education, and mean SBP (Standard BP control: n=4,134; Intensive BP control: n=4165);

Model 3: age, sex, ethnicity, education, and mean SBP, history of cardiovascular disease, stroke, current smoke, hypertension, statin use, baseline glucose, low-density lipoprotein, body mass index, depression (Standard BP control: n=3957; Intensive BP control: n=4009).

***Abbreviations:*** SBP-VIM, systolic blood pressure variation independent of mean; DSCT, Digit Symbol Coding Test; T1, tertile 1 (lowest); T2, tertile 2; T3, tertile 3 (highest); CI, confidence interval; Ref, reference; BPV, blood pressure variability; SBP, systolic blood pressure.
