# Supplementary material for: Visit‐to‐visit systolic blood pressure variability, blood pressure treatment intensity, and cognitive decline
Source: Alzheimers Dement. 2026 Jul 31;22(8):e71726. doi: 10.1002/alz.71726 (PMC13426027; doi:10.1002/alz.71726)
Supplement: Supplementary file 2 — Supporting Information [file ALZ-22-e71726-s002.pdf]

ICMJE DISCLOSURE FORM

**Date:**	6/20/2026
**Your Name:**	Wenbo Zhao
**Manuscript Title:**	Visit-to-visit systolic blood pressure variability, blood pressure treatment intensity, and cognitive decline
**Manuscript Number (if known):**	ADJ-D-26-01271

In the interest of transparency, we ask you to disclose all relationships/activities/interests listed below that are related to the content of your manuscript. "Related" means any relation with for-profit or not-for-profit third parties whose interests may be affected by the content of the manuscript. Disclosure represents a commitment to transparency and does not necessarily indicate a bias. If you are in doubt about whether to list a relationship/activity/interest, it is preferable that you do so.

The author's relationships/activities/interests should be defined broadly. For example, if your manuscript pertains to the epidemiology of hypertension, you should declare all relationships with manufacturers of antihypertensive medication, even if that medication is not mentioned in the manuscript.

In item #1 below, report all support for the work reported in this manuscript without time limit. For all other items, the time frame for disclosure is the past 36 months.

		**Name all entities with whom you have this relationship or indicate none (add rows as needed)**	**Specifications/Comments (e.g., if payments were made to you or to your institution)**
		**Time frame: Since the initial planning of the work**	
1	All support for the present manuscript (e.g., funding, provision of study materials, medical writing, article processing charges, etc.) **No time limit for this item.**	☐ None	
		National Major Science and Technology Projects of Brain Science and Brain-inspired Intelligence Young Scientist Class A	Grant 2025ZD0215802; paid to institution
		National Natural Science Fund of China	Grant 82422024; paid to institution
		British Heart Foundation	Grant number RG/F/22/110052; paid to institution
		Fundamental and Interdisciplinary Disciplines Breakthrough Plan of the Ministry of Education of China	Grant number JYB2025XDXM603; paid to institution
		The Alzheimer's Society	Grant number AS-RF-21-017; paid to institution
		Infrastructural support was provided by the Cambridge British Heart Foundation Centre of Research Excellence	Grant number RE/24/130011 (infrastructural support); paid to institution
		Cambridge University Hospitals National Institute for Health and Care Research (NIHR) Biomedical Research Centre	Grant number NIHR203312 (infrastructural support); paid to institution
		**Time frame: past 36 months**	
**2**	Grants or contracts from any entity (if not indicated in item #1 above).	☒ **None**	

		Name all entities with whom you have this relationship or indicate none (add rows as needed)	Specifications/Comments (e.g., if payments were made to you or to your institution)
**3**	Royalties or licenses	☒ **None**	
**4**	Consulting fees	☒ **None**	
**5**	Payment or honoraria for lectures, presentations, speakers bureaus, manuscript writing or educational events	☒ **None**	
**6**	Payment for expert testimony	☒ **None**	
**7**	Support for attending meetings and/or travel	☒ **None**	
**8**	Patents planned, issued or pending	☒ **None**	
**9**	Participation on a Data Safety Monitoring Board or Advisory Board	☒ **None**	
**10**	Leadership or fiduciary role in other board,	☒ **None**	

		Name all entities with whom you have this relationship or indicate none (add rows as needed)	Specifications/Comments (e.g., if payments were made to you or to your institution)
	society, committee or advocacy group, paid or unpaid		
**11**	Stock or stock options	☒ **None**	
**12**	Receipt of equipment, materials, drugs, medical writing, gifts or other services	☒ **None**	
**13**	Other financial or non-financial interests	☒ **None**	

Please place an "X" next to the following statement to indicate your agreement:

☒ I certify that I have answered every question and have not altered the wording of any of the questions on this form.

ICMJE DISCLOSURE FORM

**Date:**	6/20/2026
**Your Name:**	Yue Qiao
**Manuscript Title:**	Visit-to-visit systolic blood pressure variability, blood pressure treatment intensity, and cognitive decline
**Manuscript Number (if known):**	ADJ-D-26-01271

In the interest of transparency, we ask you to disclose all relationships/activities/interests listed below that are related to the content of your manuscript. "Related" means any relation with for-profit or not-for-profit third parties whose interests may be affected by the content of the manuscript. Disclosure represents a commitment to transparency and does not necessarily indicate a bias. If you are in doubt about whether to list a relationship/activity/interest, it is preferable that you do so.

The author's relationships/activities/interests should be defined broadly. For example, if your manuscript pertains to the epidemiology of hypertension, you should declare all relationships with manufacturers of antihypertensive medication, even if that medication is not mentioned in the manuscript.

In item #1 below, report all support for the work reported in this manuscript without time limit. For all other items, the time frame for disclosure is the past 36 months.

		**Name all entities with whom you have this relationship or indicate none (add rows as needed)**	**Specifications/Comments (e.g., if payments were made to you or to your institution)**
		Time frame: Since the initial planning of the work	
1	All support for the present manuscript (e.g., funding, provision of study materials, medical writing, article processing charges, etc.) **No time limit for this item.**	☐ None	
		National Major Science and Technology Projects of Brain Science and Brain-inspired Intelligence Young Scientist Class A	Grant 2025ZD0215802; paid to institution
		National Natural Science Fund of China	Grant 82422024; paid to institution
		British Heart Foundation	Grant number RG/F/22/110052; paid to institution
		Fundamental and Interdisciplinary Disciplines Breakthrough Plan of the Ministry of Education of China	Grant number JYB2025XDXM603; paid to institution
		The Alzheimer's Society	Grant number AS-RF-21-017; paid to institution
		Infrastructural support was provided by the Cambridge British Heart Foundation Centre of Research Excellence	Grant number RE/24/130011 (infrastructural support); paid to institution
		Cambridge University Hospitals National Institute for Health and Care Research (NIHR) Biomedical Research Centre	Grant number NIHR203312 (infrastructural support); paid to institution
		Time frame: past 36 months	
**2**	Grants or contracts from any entity (if not indicated in item #1 above).	☒ **None**	

		Name all entities with whom you have this relationship or indicate none (add rows as needed)	Specifications/Comments (e.g., if payments were made to you or to your institution)
**3**	Royalties or licenses	☒ **None**	
**4**	Consulting fees	☒ **None**	
**5**	Payment or honoraria for lectures, presentations, speakers bureaus, manuscript writing or educational events	☒ **None**	
**6**	Payment for expert testimony	☒ **None**	
**7**	Support for attending meetings and/or travel	☒ **None**	
**8**	Patents planned, issued or pending	☒ **None**	
**9**	Participation on a Data Safety Monitoring Board or Advisory Board	☒ **None**	
**10**	Leadership or fiduciary role in other board,	☒ **None**	

		Name all entities with whom you have this relationship or indicate none (add rows as needed)	Specifications/Comments (e.g., if payments were made to you or to your institution)
	society, committee or advocacy group, paid or unpaid		
**11**	Stock or stock options	☒ **None**	
**12**	Receipt of equipment, materials, drugs, medical writing, gifts or other services	☒ **None**	
**13**	Other financial or non-financial interests	☒ **None**	

Please place an "X" next to the following statement to indicate your agreement:

☒ I certify that I have answered every question and have not altered the wording of any of the questions on this form.

ICMJE DISCLOSURE FORM

Date: 6/20/2026

Your Name: Zihan Sun

Manuscript Title: Visit-to-visit systolic blood pressure variability, blood pressure treatment intensity, and cognitive decline

Manuscript Number (if known): ADJ-D-26-01271

In the interest of transparency, we ask you to disclose all relationships/activities/interests listed below that are related to the content of your manuscript. "Related" means any relation with for-profit or not-for-profit third parties whose interests may be affected by the content of the manuscript. Disclosure represents a commitment to transparency and does not necessarily indicate a bias. If you are in doubt about whether to list a relationship/activity/interest, it is preferable that you do so.

The author's relationships/activities/interests should be defined broadly. For example, if your manuscript pertains to the epidemiology of hypertension, you should declare all relationships with manufacturers of antihypertensive medication, even if that medication is not mentioned in the manuscript.

In item #1 below, report all support for the work reported in this manuscript without time limit. For all other items, the time frame for disclosure is the past 36 months.

		**Name all entities with whom you have this relationship or indicate none (add rows as needed)**	**Specifications/Comments (e.g., if payments were made to you or to your institution)**
**Time frame: Since the initial planning of the work**			
1	All support for the present manuscript (e.g., funding, provision of study materials, medical writing, article processing charges, etc.) **No time limit for this item.**	☐ None	
		National Major Science and Technology Projects of Brain Science and Brain-inspired Intelligence Young Scientist Class A	Grant 2025ZD0215802; paid to institution
		National Natural Science Fund of China	Grant 82422024; paid to institution
		British Heart Foundation	Grant number RG/F/22/110052; paid to institution
		Fundamental and Interdisciplinary Disciplines Breakthrough Plan of the Ministry of Education of China	Grant number JYB2025XDXM603; paid to institution
		The Alzheimer's Society	Grant number AS-RF-21-017; paid to institution
		Infrastructural support was provided by the Cambridge British Heart Foundation Centre of Research Excellence	Grant number RE/24/130011 (infrastructural support); paid to institution
		Cambridge University Hospitals National Institute for Health and Care Research (NIHR) Biomedical Research Centre	Grant number NIHR203312 (infrastructural support); paid to institution
**Time frame: past 36 months**			
2	Grants or contracts from any entity (if not indicated in item #1 above).	☒ **None**	

		Name all entities with whom you have this relationship or indicate none (add rows as needed)	Specifications/Comments (e.g., if payments were made to you or to your institution)
**3**	Royalties or licenses	☒ **None**	
**4**	Consulting fees	☒ **None**	
**5**	Payment or honoraria for lectures, presentations, speakers bureaus, manuscript writing or educational events	☒ **None**	
**6**	Payment for expert testimony	☒ **None**	
**7**	Support for attending meetings and/or travel	☒ **None**	
**8**	Patents planned, issued or pending	☒ **None**	
**9**	Participation on a Data Safety Monitoring Board or Advisory Board	☒ **None**	
**10**	Leadership or fiduciary role in other board,	☒ **None**	

		Name all entities with whom you have this relationship or indicate none (add rows as needed)	Specifications/Comments (e.g., if payments were made to you or to your institution)
	society, committee or advocacy group, paid or unpaid		
**11**	Stock or stock options	☒ **None**	
**12**	Receipt of equipment, materials, drugs, medical writing, gifts or other services	☒ **None**	
**13**	Other financial or non-financial interests	☒ **None**	

Please place an "X" next to the following statement to indicate your agreement:

☒ I certify that I have answered every question and have not altered the wording of any of the questions on this form.

ICMJE DISCLOSURE FORM

**Date:**	6/20/2026
**Your Name:**	Xunming Ji
**Manuscript Title:**	Visit-to-visit systolic blood pressure variability, blood pressure treatment intensity, and cognitive decline
**Manuscript Number (if known):**	ADJ-D-26-01271

In the interest of transparency, we ask you to disclose all relationships/activities/interests listed below that are related to the content of your manuscript. "Related" means any relation with for-profit or not-for-profit third parties whose interests may be affected by the content of the manuscript. Disclosure represents a commitment to transparency and does not necessarily indicate a bias. If you are in doubt about whether to list a relationship/activity/interest, it is preferable that you do so.

The author's relationships/activities/interests should be defined broadly. For example, if your manuscript pertains to the epidemiology of hypertension, you should declare all relationships with manufacturers of antihypertensive medication, even if that medication is not mentioned in the manuscript.

In item #1 below, report all support for the work reported in this manuscript without time limit. For all other items, the time frame for disclosure is the past 36 months.

		**Name all entities with whom you have this relationship or indicate none (add rows as needed)**	**Specifications/Comments (e.g., if payments were made to you or to your institution)**
**Time frame: Since the initial planning of the work**			
1	All support for the present manuscript (e.g., funding, provision of study materials, medical writing, article processing charges, etc.) **No time limit for this item.**	☐ None	
		National Major Science and Technology Projects of Brain Science and Brain-inspired Intelligence Young Scientist Class A	Grant 2025ZD0215802; paid to institution
		National Natural Science Fund of China	Grant 82422024; paid to institution
		British Heart Foundation	Grant number RG/F/22/110052; paid to institution
		Fundamental and Interdisciplinary Disciplines Breakthrough Plan of the Ministry of Education of China	Grant number JYB2025XDXM603; paid to institution
		The Alzheimer's Society	Grant number AS-RF-21-017; paid to institution
		Infrastructural support was provided by the Cambridge British Heart Foundation Centre of Research Excellence	Grant number RE/24/130011 (infrastructural support); paid to institution
		Cambridge University Hospitals National Institute for Health and Care Research (NIHR) Biomedical Research Centre	Grant number NIHR203312 (infrastructural support); paid to institution
**Time frame: past 36 months**			
**2**	Grants or contracts from any entity (if not indicated in item #1 above).	☒ **None**	

		Name all entities with whom you have this relationship or indicate none (add rows as needed)	Specifications/Comments (e.g., if payments were made to you or to your institution)
**3**	Royalties or licenses	☒ **None**	
**4**	Consulting fees	☒ **None**	
**5**	Payment or honoraria for lectures, presentations, speakers bureaus, manuscript writing or educational events	☒ **None**	
**6**	Payment for expert testimony	☒ **None**	
**7**	Support for attending meetings and/or travel	☒ **None**	
**8**	Patents planned, issued or pending	☒ **None**	
**9**	Participation on a Data Safety Monitoring Board or Advisory Board	☒ **None**	
**10**	Leadership or fiduciary role in other board,	☒ **None**	

		Name all entities with whom you have this relationship or indicate none (add rows as needed)	Specifications/Comments (e.g., if payments were made to you or to your institution)
	society, committee or advocacy group, paid or unpaid		
**11**	Stock or stock options	☒ **None**	
**12**	Receipt of equipment, materials, drugs, medical writing, gifts or other services	☒ **None**	
**13**	Other financial or non-financial interests	☒ **None**	

Please place an "X" next to the following statement to indicate your agreement:

☒ I certify that I have answered every question and have not altered the wording of any of the questions on this form.

ICMJE DISCLOSURE FORM

**Date:**	6/20/2026
**Your Name:**	Hugh S Markus
**Manuscript Title:**	Visit-to-visit systolic blood pressure variability, blood pressure treatment intensity, and cognitive decline
**Manuscript Number (if known):**	ADJ-D-26-01271

In the interest of transparency, we ask you to disclose all relationships/activities/interests listed below that are related to the content of your manuscript. "Related" means any relation with for-profit or not-for-profit third parties whose interests may be affected by the content of the manuscript. Disclosure represents a commitment to transparency and does not necessarily indicate a bias. If you are in doubt about whether to list a relationship/activity/interest, it is preferable that you do so.

The author's relationships/activities/interests should be defined broadly. For example, if your manuscript pertains to the epidemiology of hypertension, you should declare all relationships with manufacturers of antihypertensive medication, even if that medication is not mentioned in the manuscript.

In item #1 below, report all support for the work reported in this manuscript without time limit. For all other items, the time frame for disclosure is the past 36 months.

		**Name all entities with whom you have this relationship or indicate none (add rows as needed)**	**Specifications/Comments (e.g., if payments were made to you or to your institution)**
		Time frame: Since the initial planning of the work	
1	All support for the present manuscript (e.g., funding, provision of study materials, medical writing, article processing charges, etc.) **No time limit for this item.**	☐ None	
		National Major Science and Technology Projects of Brain Science and Brain-inspired Intelligence Young Scientist Class A	Grant 2025ZD0215802; paid to institution
		National Natural Science Fund of China	Grant 82422024; paid to institution
		British Heart Foundation	Grant number RG/F/22/110052; paid to institution
		Fundamental and Interdisciplinary Disciplines Breakthrough Plan of the Ministry of Education of China	Grant number JYB2025XDXM603; paid to institution
		The Alzheimer's Society	Grant number AS-RF-21-017; paid to institution
		Infrastructural support was provided by the Cambridge British Heart Foundation Centre of Research Excellence	Grant number RE/24/130011 (infrastructural support); paid to institution
		Cambridge University Hospitals National Institute for Health and Care Research (NIHR) Biomedical Research Centre	Grant number NIHR203312 (infrastructural support); paid to institution
		Time frame: past 36 months	
2	Grants or contracts from any entity (if not indicated in item #1 above).	☒ **None**	

		Name all entities with whom you have this relationship or indicate none (add rows as needed)	Specifications/Comments (e.g., if payments were made to you or to your institution)
**3**	Royalties or licenses	☒ **None**	
**4**	Consulting fees	☒ **None**	
**5**	Payment or honoraria for lectures, presentations, speakers bureaus, manuscript writing or educational events	☒ **None**	
**6**	Payment for expert testimony	☒ **None**	
**7**	Support for attending meetings and/or travel	☒ **None**	
**8**	Patents planned, issued or pending	☒ **None**	
**9**	Participation on a Data Safety Monitoring Board or Advisory Board	☒ **None**	
**10**	Leadership or fiduciary role in other board,	☒ **None**	

		Name all entities with whom you have this relationship or indicate none (add rows as needed)	Specifications/Comments (e.g., if payments were made to you or to your institution)
	society, committee or advocacy group, paid or unpaid		
**11**	Stock or stock options	☒ **None**	
**12**	Receipt of equipment, materials, drugs, medical writing, gifts or other services	☒ **None**	
**13**	Other financial or non-financial interests	☒ **None**	

Please place an "X" next to the following statement to indicate your agreement:

☒ I certify that I have answered every question and have not altered the wording of any of the questions on this form.
